# Supplementary material for: Structural determinants of dynamic fluctuations between segregation and integration on the human connectome
Source: Commun Biol. 2020 Oct 23;3:606. doi: 10.1038/s42003-020-01331-3 (PMC7584581; doi:10.1038/s42003-020-01331-3)
Supplement: Supplementary file 1 — Supplementary Information [file 42003_2020_1331_MOESM1_ESM.pdf]

Supplementary Information for

Structural determinants of dynamic fluctuations between segregation and integration on the human connectome

Makoto Fukushima*, Olaf Sporns

*Corresponding author at Division of Information Science, Graduate School of Science and Technology, Nara Institute of Science and Technology, 8916-5 Takayama-cho, Ikoma, Nara, 630-0192, Japan

Email address: mfukushi@is.naist.jp

Supplementary Figures

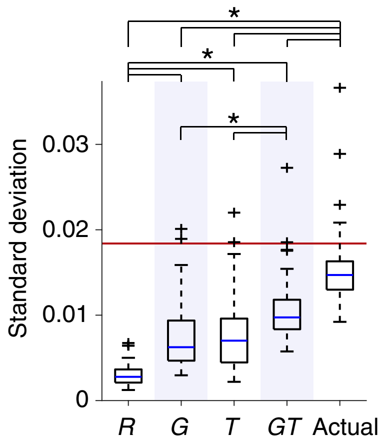

Supplementary Fig. 1 The SD of dynamic fluctuations in mean TPC. The median SD computed from empirical rs-fMRI data is shown by a red vertical line. An asterisk indicates significant differences between the SDs ($p < 0.05$, FDR corrected across all the 10 comparisons). The exact p and n are shown in Supplementary Table 2.

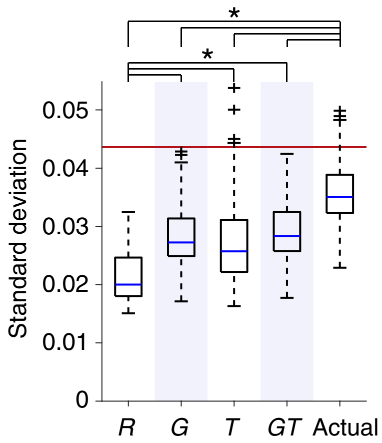

Supplementary Fig. 2 The SD of dynamic fluctuations in modularity. The median SD computed from empirical rs-fMRI data is shown by a red vertical line. An asterisk indicates significant differences between the SDs ($p < 0.05$, FDR corrected across all the 10 comparisons). The exact p and n are shown in Supplementary Table 3.

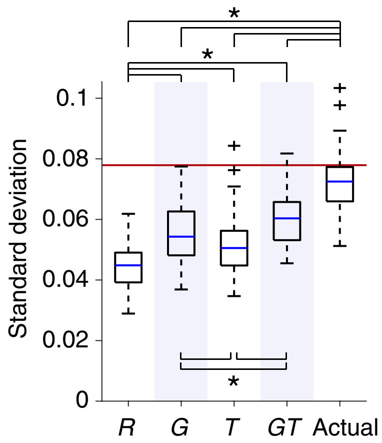

Supplementary Fig. 3 The SD of dynamic fluctuations in mean PC with a window width of 44 TRs. The median SD computed from empirical rs-fMRI data is shown by a red vertical line. An asterisk indicates significant differences between the SDs ($p < 0.05$, FDR corrected across all the 10 comparisons). The exact p and n are shown in Supplementary Table 4.

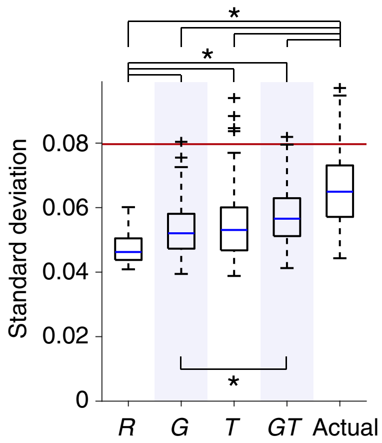

Supplementary Fig. 4 The SD of dynamic fluctuations in mean PC with a window width of 88 TRs. The median SD computed from empirical rs-fMRI data is shown by a red vertical line. An asterisk indicates significant differences between the SDs ($p < 0.05$, FDR corrected across all the 10 comparisons). The exact p and n are shown in Supplementary Table 5.

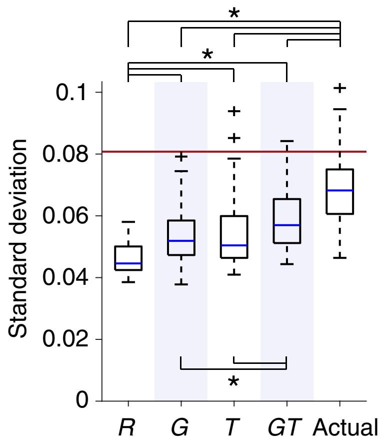

Supplementary Fig. 5 The SD of dynamic fluctuations in mean PC with a window displacement of 1 TR. The median SD computed from empirical rs-fMRI data is shown by a red vertical line. An asterisk indicates significant differences between the SDs ($p < 0.05$, FDR corrected across all the 10 comparisons). The exact p and n are shown in Supplementary Table 6.

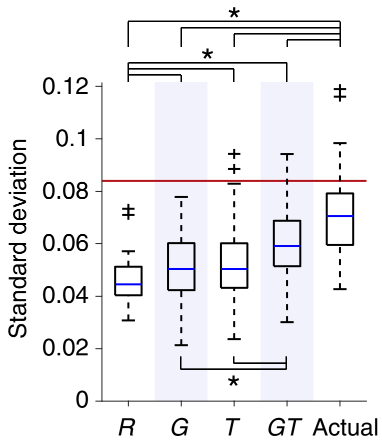

Supplementary Fig. 6 The SD of dynamic fluctuations in mean PC with a window displacement of 66 TRs. The median SD computed from empirical rs-fMRI data is shown by a red vertical line. An asterisk indicates significant differences between the SDs ($p < 0.05$, FDR corrected across all the 10 comparisons). The exact p and n are shown in Supplementary Table 7.

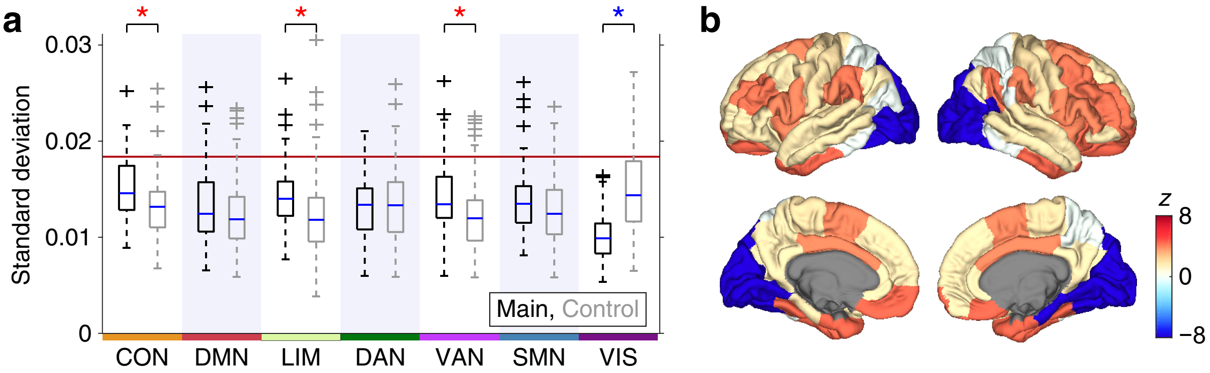

Supplementary Fig. 7 The SD of dynamic fluctuations in mean TPC, obtained from each main–control pair of the RSN-constrained surrogate connectome data. **a** The SD of mean TPC across time. The median SD computed from empirical rs-fMRI data is shown by a red vertical line. An asterisk indicates significant differences between the SDs ($p < 0.05$, FDR corrected across all the 7 comparisons; blue asterisk, Main < Control; red asterisk, Main > Control). The exact p and n are shown in Supplementary Table 9. **b** Cortical surface plots of the z-values of differences between the SDs (Main − Control).

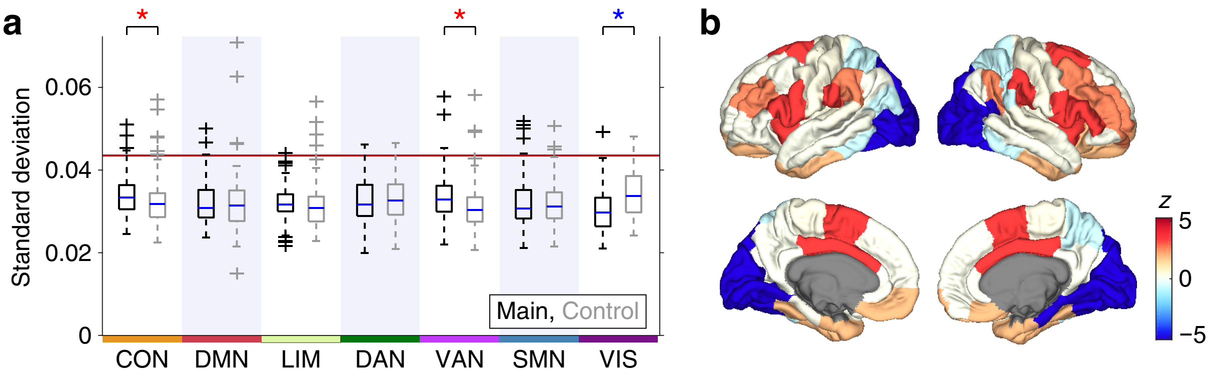

Supplementary Fig. 8 The SD of dynamic fluctuations in modularity, obtained from each main–control pair of the RSN-constrained surrogate connectome data. **a** The SD of modularity across time. The median SD computed from empirical rs-fMRI data is shown by a red vertical line. An asterisk indicates significant differences between the SDs ($p < 0.05$, FDR corrected across all the 7 comparisons; blue asterisk, Main $<$ Control; red asterisk, Main $>$ Control). The exact p and n are shown in Supplementary Table 10. **b** Cortical surface plots of the z-values of differences between the SDs (Main $-$ Control).

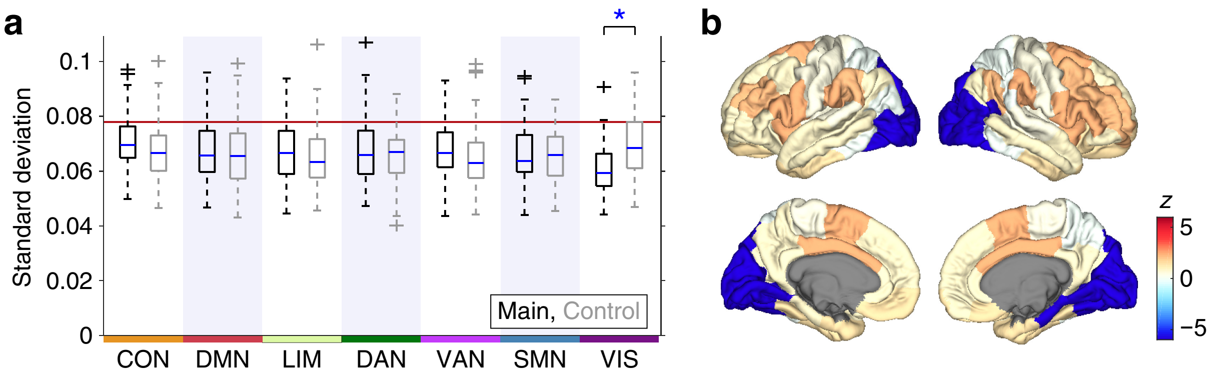

Supplementary Fig. 9 The SD of dynamic fluctuations in mean PC with a window width of 44 TRs, obtained from each main–control pair of the RSN-constrained surrogate connectome data. **a** The SD of mean PC across time. The median SD computed from empirical rs-fMRI data is shown by a red vertical line. An asterisk indicates significant differences between the SDs ($p < 0.05$, FDR corrected across all the 7 comparisons). The exact p and n are shown in Supplementary Table 11. **b** Cortical surface plots of the z-values of differences between the SDs (Main − Control).

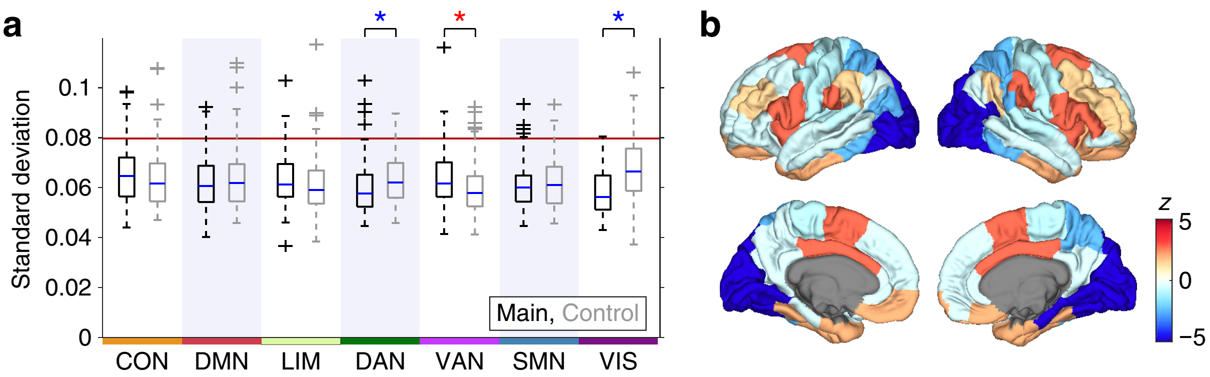

Supplementary Fig. 10 The SD of dynamic fluctuations in mean PC with a window width of 88 TRs, obtained from each main–control pair of the RSN-constrained surrogate connectome data. **a** The SD of mean PC across time. The median SD computed from empirical rs-fMRI data is shown by a red vertical line. An asterisk indicates significant differences between the SDs ($p < 0.05$, FDR corrected across all the 7 comparisons; blue asterisk, Main < Control; red asterisk, Main > Control). The exact p and n are shown in Supplementary Table 12. **b** Cortical surface plots of the z-values of differences between the SDs (Main − Control).

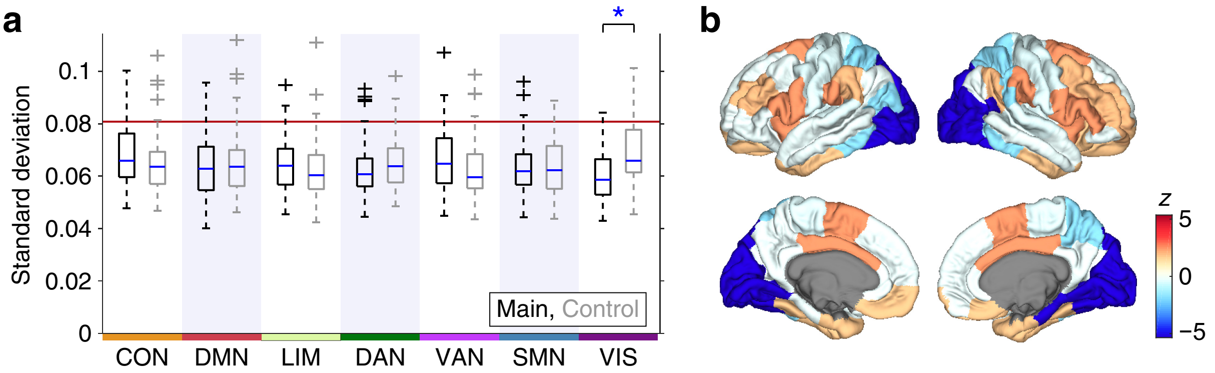

Supplementary Fig. 11 The SD of dynamic fluctuations in mean PC with a window displacement of 1 TR, obtained from each main–control pair of the RSN-constrained surrogate connectome data. **a** The SD of mean PC across time. The median SD computed from empirical rs-fMRI data is shown by a red vertical line. An asterisk indicates significant differences between the SDs ($p < 0.05$, FDR corrected across all the 7 comparisons). The exact p and n are shown in Supplementary Table 13. **b** Cortical surface plots of the z-values of differences between the SDs (Main − Control).

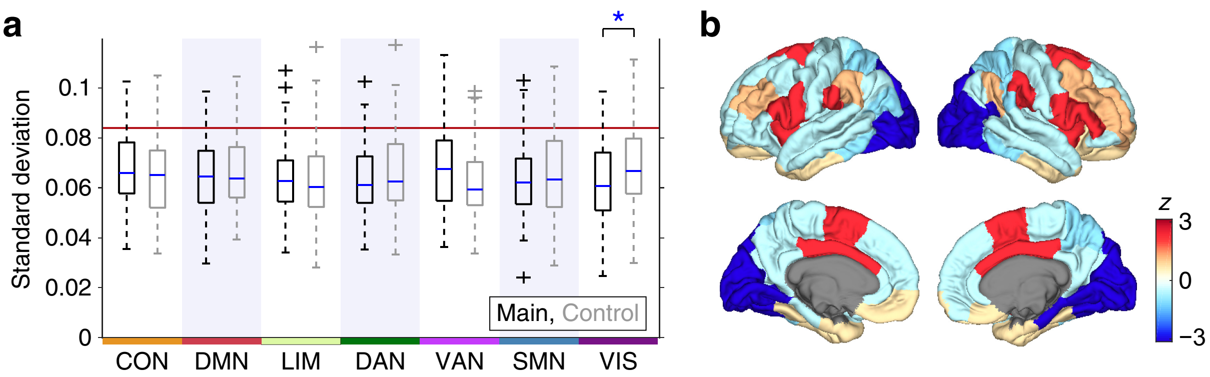

Supplementary Fig. 12 The SD of dynamic fluctuations in mean PC with a window displacement of 66 TRs, obtained from each main–control pair of the RSN-constrained surrogate connectome data. **a** The SD of mean PC across time. The median SD computed from empirical rs-fMRI data is shown by a red vertical line. An asterisk indicates significant differences between the SDs ($p < 0.05$, FDR corrected across all the 7 comparisons). The exact p and n are shown in Supplementary Table 14. **b** Cortical surface plots of the z-values of differences between the SDs (Main − Control).

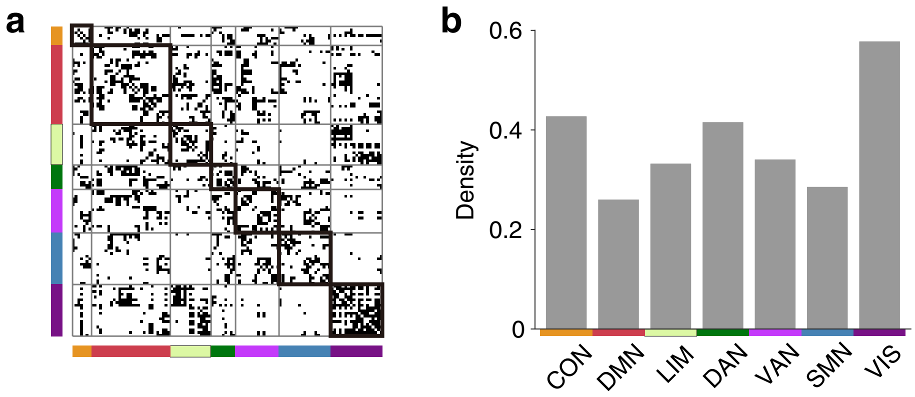

Supplementary Fig. 13 The density of structural connections. **a** Structural connectivity (binarized) sorted based on the assignment of nodes to the seven RSNs. **b** The density of structural connections within each RSN.

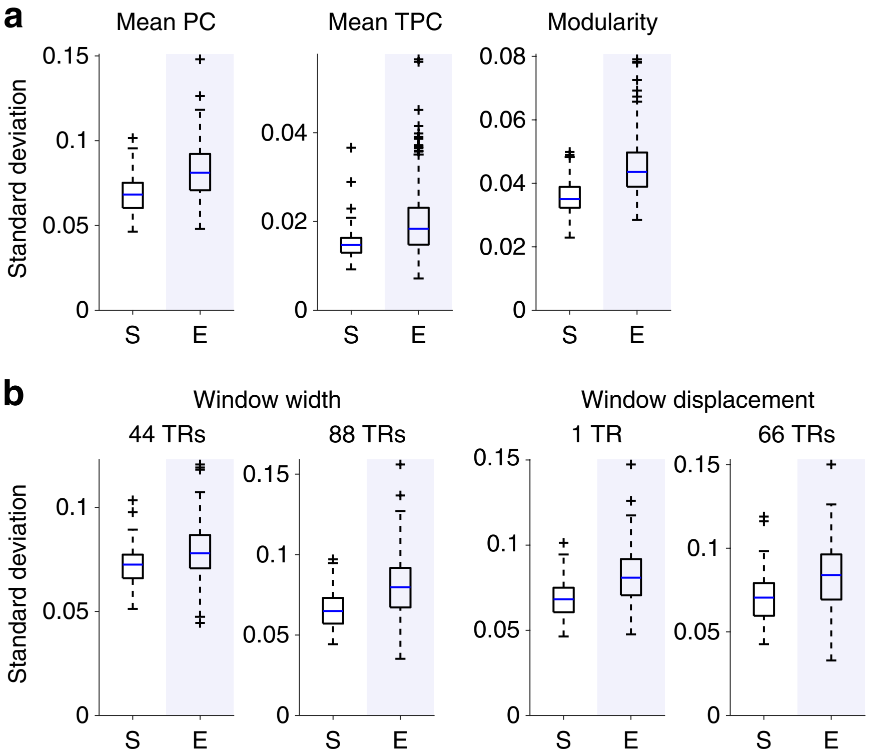

Supplementary Fig. 14 The SD of dynamic fluctuations in global network measures for segregation and integration. **a** The SD of dynamic fluctuations in mean PC, mean TPC, and modularity (S: simulated data with the actual connectome; E: empirical data). **b** The SD of dynamic fluctuations in mean PC with different window parameters. The exact p and n are shown in Supplementary Table 15.

Supplementary Tables

Supplementary Table 1. Comparison of the SD of dynamic fluctuations in mean PC among the surrogate and actual connectome data (Mann-Whitney U test). $p < 0.05$ (two-sided, FDR corrected) is shown in **bold**.

	${}^{1}G - {}^{2}R$	${}^{1}T - {}^{2}R$	${}^{1}GT - {}^{2}R$	${}^{1}T - {}^{2}G$	${}^{1}GT - {}^{2}G$	${}^{1}GT - {}^{2}T$
p	$\mathbf{3.8 \times 10^{-6}}$	$\mathbf{7.3 \times 10^{-6}}$	$\mathbf{4.0 \times 10^{-11}}$	0.83	$\mathbf{4.9 \times 10^{-3}}$	$\mathbf{8.2 \times 10^{-3}}$
median_1	0.052	0.051	0.057	0.051	0.057	0.057
median_2	0.045	0.045	0.045	0.052	0.052	0.051
n_1	50	50	50	50	50	50
n_2	50	50	50	50	50	50
U	1936	1912	2234	1219	1669	1639
z-value	4.7	4.6	6.8	−0.21	2.9	2.7
Cliff's delta	0.55	0.53	0.79	−0.025	0.34	0.31
95% CI of delta	[0.34, 0.70]	[0.32, 0.69]	[0.62, 0.88]	[−0.24, 0.20]	[0.11, 0.52]	[0.08, 0.50]

	${}^{1}\text{Actual} - {}^{2}R$	${}^{1}\text{Actual} - {}^{2}G$	${}^{1}\text{Actual} - {}^{2}T$	${}^{1}\text{Actual} - {}^{2}GT$
p	$\mathbf{7.8 \times 10^{-21}}$	$\mathbf{1.4 \times 10^{-12}}$	$\mathbf{1.4 \times 10^{-10}}$	$\mathbf{3.9 \times 10^{-7}}$
median_1	0.068	0.068	0.068	0.068
median_2	0.045	0.052	0.051	0.057
n_1	100	100	100	100
n_2	50	50	50	50
U	4909	4333	4145	3806
z-value	9.6	7.3	6.6	5.2
Cliff's delta	0.96	0.73	0.66	0.52
95% CI of delta	[0.88, 0.99]	[0.58, 0.83]	[0.49, 0.77]	[0.34, 0.66]

Supplementary Table 2. Comparison of the SD of dynamic fluctuations in mean TPC among the surrogate and actual connectome data (Mann-Whitney U test). $p < 0.05$ (two-sided, FDR corrected) is shown in **bold**.

	${}^1G - {}^2R$	${}^1T - {}^2R$	${}^1GT - {}^2R$	${}^1T - {}^2G$	${}^1GT - {}^2G$	${}^1GT - {}^2T$
p	$\mathbf{8.6 \times 10^{-13}}$	$\mathbf{4.9 \times 10^{-12}}$	$\mathbf{1.0 \times 10^{-16}}$	0.86	$\mathbf{1.6 \times 10^{-5}}$	$\mathbf{7.0 \times 10^{-5}}$
median_1	6.3×10^{-3}	7.0×10^{-3}	9.7×10^{-3}	7.0×10^{-3}	9.7×10^{-3}	9.7×10^{-3}
median_2	2.8×10^{-3}	2.8×10^{-3}	2.8×10^{-3}	6.3×10^{-3}	6.3×10^{-3}	7.0×10^{-3}
n_1	50	50	50	50	50	50
n_2	50	50	50	50	50	50
U	2298	2260	2482	1276	1883	1831
z-value	7.2	7.0	8.5	0.18	4.4	4.0
Cliff's delta	0.84	0.81	0.99	0.021	0.51	0.46
95% CI of delta	$[0.68, 0.92]$	$[0.64, 0.90]$	$[0.89, 1.00]$	$[-0.20, 0.24]$	$[0.29, 0.67]$	$[0.25, 0.63]$

	${}^1\text{Actual} - {}^2R$	${}^1\text{Actual} - {}^2G$	${}^1\text{Actual} - {}^2T$	${}^1\text{Actual} - {}^2GT$
p	$\mathbf{2.2 \times 10^{-22}}$	$\mathbf{3.8 \times 10^{-16}}$	$\mathbf{4.8 \times 10^{-15}}$	$\mathbf{6.0 \times 10^{-13}}$
median_1	0.015	0.015	0.015	0.015
median_2	2.8×10^{-3}	6.3×10^{-3}	7.0×10^{-3}	9.7×10^{-3}
n_1	100	100	100	100
n_2	50	50	50	50
U	5000	4580	4494	4330
z-value	10	8.3	7.9	7.3
Cliff's delta	1.0	0.83	0.80	0.73
95% CI of delta	$[0.94, 1.00]$	$[0.70, 0.91]$	$[0.66, 0.88]$	$[0.58, 0.83]$

Supplementary Table 3. Comparison of the SD of dynamic fluctuations in modularity among the surrogate and actual connectome data (Mann-Whitney U test). $p < 0.05$ (two-sided, FDR corrected) is shown in **bold**.

	$^1G - {}^2R$	$^1T - {}^2R$	$^1GT - {}^2R$	$^1T - {}^2G$	$^1GT - {}^2G$	$^1GT - {}^2T$
p	$\mathbf{5.5 \times 10^{-10}}$	$\mathbf{7.6 \times 10^{-7}}$	$\mathbf{5.5 \times 10^{-11}}$	0.17	0.39	0.060
median_1	0.027	0.026	0.028	0.026	0.028	0.028
median_2	0.020	0.020	0.020	0.027	0.027	0.026
n_1	50	50	50	50	50	50
n_2	50	50	50	50	50	50
U	2162	1978	2236	1040	1374	1537
z-value	6.3	5.0	6.8	−1.4	0.85	2.0
Cliff's delta	0.73	0.58	0.79	−0.17	0.099	0.23
95% CI of delta	[0.55, 0.84]	[0.37, 0.73]	[0.62, 0.88]	[−0.38, 0.06]	[−0.13, 0.31]	[0.00, 0.43]

	$^1\text{Actual} - {}^2R$	$^1\text{Actual} - {}^2G$	$^1\text{Actual} - {}^2T$	$^1\text{Actual} - {}^2GT$
p	$\mathbf{4.1 \times 10^{-21}}$	$\mathbf{8.1 \times 10^{-11}}$	$\mathbf{1.3 \times 10^{-10}}$	$\mathbf{6.1 \times 10^{-11}}$
median_1	0.035	0.035	0.035	0.035
median_2	0.020	0.027	0.026	0.028
n_1	100	100	100	100
n_2	50	50	50	50
U	4926	4165	4138	4186
z-value	9.7	6.6	6.5	6.7
Cliff's delta	0.97	0.67	0.66	0.67
95% CI of delta	[0.89, 0.99]	[0.50, 0.78]	[0.49, 0.77]	[0.51, 0.79]

Supplementary Table 4. Comparison of the SD of dynamic fluctuations in mean PC with a window width of 44 TRs among the surrogate and actual connectome data (Mann-Whitney U test). $p < 0.05$ ($z > 0$) and $p < 0.05$ ($z < 0$) (two-sided, FDR corrected) are shown in **bold** and ***bold italic***, respectively.

	$^1G - {}^2R$	$^1T - {}^2R$	$^1GT - {}^2R$	$^1T - {}^2G$	$^1GT - {}^2G$	$^1GT - {}^2T$
p	$\mathbf{4.0 \times 10^{-8}}$	$\mathbf{2.6 \times 10^{-4}}$	$\mathbf{6.2 \times 10^{-13}}$	***0.021***	**0.021**	$\mathbf{7.5 \times 10^{-6}}$
median_1	0.054	0.051	0.060	0.051	0.060	0.060
median_2	0.045	0.045	0.045	0.054	0.054	0.051
n_1	50	50	50	50	50	50
n_2	50	50	50	50	50	50
U	2060	1788	2318	915	1587	1911
z-value	5.6	3.7	7.4	−2.3	2.3	4.6
Cliff's delta	0.65	0.43	0.85	−0.27	0.27	0.53
95% CI of delta	[0.45, 0.78]	[0.21, 0.60]	[0.70, 0.93]	[−0.46, −0.04]	[0.04, 0.47]	[0.32, 0.69]

	$^1\text{Actual} - {}^2R$	$^1\text{Actual} - {}^2G$	$^1\text{Actual} - {}^2T$	$^1\text{Actual} - {}^2GT$
p	$\mathbf{1.1 \times 10^{-21}}$	$\mathbf{1.0 \times 10^{-12}}$	$\mathbf{3.0 \times 10^{-16}}$	$\mathbf{3.0 \times 10^{-9}}$
median_1	0.073	0.073	0.073	0.073
median_2	0.045	0.054	0.051	0.060
n_1	100	100	100	100
n_2	50	50	50	50
U	4959	4320	4599	4017
z-value	9.8	7.3	8.4	6.0
Cliff's delta	0.98	0.73	0.84	0.61
95% CI of delta	[0.91, 1.00]	[0.57, 0.83]	[0.71, 0.91]	[0.43, 0.73]

Supplementary Table 5. Comparison of the SD of dynamic fluctuations in mean PC with a window width of 88 TRs among the surrogate and actual connectome data (Mann-Whitney U test). $p < 0.05$ (two-sided, FDR corrected) is shown in **bold**.

	$^1G - {}^2R$	$^1T - {}^2R$	$^1GT - {}^2R$	$^1T - {}^2G$	$^1GT - {}^2G$	$^1GT - {}^2T$
p	$\mathbf{6.3 \times 10^{-4}}$	$\mathbf{5.3 \times 10^{-5}}$	$\mathbf{2.2 \times 10^{-8}}$	0.51	**0.022**	0.17
median_1	0.052	0.053	0.057	0.053	0.057	0.057
median_2	0.046	0.046	0.046	0.052	0.052	0.053
n_1	50	50	50	50	50	50
n_2	50	50	50	50	50	50
U	1760	1860	2092	1347	1595	1458
z-value	3.5	4.2	5.8	0.67	2.4	1.4
Cliff's delta	0.41	0.49	0.67	0.078	0.28	0.17
95% CI of delta	$[0.19, 0.59]$	$[0.27, 0.65]$	$[0.48, 0.80]$	$[-0.15, 0.29]$	$[0.05, 0.47]$	$[-0.06, 0.37]$

	$^1\text{Actual} - {}^2R$	$^1\text{Actual} - {}^2G$	$^1\text{Actual} - {}^2T$	$^1\text{Actual} - {}^2GT$
p	$\mathbf{3.4 \times 10^{-18}}$	$\mathbf{4.5 \times 10^{-9}}$	$\mathbf{7.4 \times 10^{-7}}$	$\mathbf{1.0 \times 10^{-4}}$
median_1	0.065	0.065	0.065	0.065
median_2	0.046	0.052	0.053	0.057
n_1	100	100	100	100
n_2	50	50	50	50
U	4747	4037	3786	3505
z-value	9.0	6.1	5.1	4.0
Cliff's delta	0.90	0.61	0.51	0.40
95% CI of delta	$[0.79, 0.95]$	$[0.44, 0.74]$	$[0.33, 0.66]$	$[0.21, 0.56]$

Supplementary Table 6. Comparison of the SD of dynamic fluctuations in mean PC with a window displacement of 1 TR among the surrogate and actual connectome data (Mann-Whitney U test). $p < 0.05$ (two-sided, FDR corrected) is shown in **bold**.

	${}^1G - {}^2R$	${}^1T - {}^2R$	${}^1GT - {}^2R$	${}^1T - {}^2G$	${}^1GT - {}^2G$	${}^1GT - {}^2T$
p	$\mathbf{2.9 \times 10^{-6}}$	$\mathbf{6.8 \times 10^{-6}}$	$\mathbf{1.6 \times 10^{-11}}$	0.81	$\mathbf{4.3 \times 10^{-3}}$	$\mathbf{7.6 \times 10^{-3}}$
median_1	0.052	0.050	0.057	0.050	0.057	0.057
median_2	0.045	0.045	0.045	0.052	0.052	0.050
n_1	50	50	50	50	50	50
n_2	50	50	50	50	50	50
U	1944	1914	2253	1215	1675	1643
z-value	4.8	4.6	6.9	-0.24	2.9	2.7
Cliff's delta	0.56	0.53	0.80	-0.028	0.34	0.31
95% CI of delta	$[0.34, 0.71]$	$[0.32, 0.69]$	$[0.63, 0.89]$	$[-0.25, 0.19]$	$[0.11, 0.53]$	$[0.09, 0.51]$

	${}^1\text{Actual} - {}^2R$	${}^1\text{Actual} - {}^2G$	${}^1\text{Actual} - {}^2T$	${}^1\text{Actual} - {}^2GT$
p	$\mathbf{8.8 \times 10^{-21}}$	$\mathbf{1.1 \times 10^{-12}}$	$\mathbf{1.8 \times 10^{-10}}$	$\mathbf{7.7 \times 10^{-7}}$
median_1	0.068	0.068	0.068	0.068
median_2	0.045	0.052	0.050	0.057
n_1	100	100	100	100
n_2	50	50	50	50
U	4906	4342	4134	3774
z-value	9.6	7.3	6.5	5.1
Cliff's delta	0.96	0.74	0.65	0.51
95% CI of delta	$[0.88, 0.99]$	$[0.58, 0.84]$	$[0.49, 0.77]$	$[0.33, 0.65]$

Supplementary Table 7. Comparison of the SD of dynamic fluctuations in mean PC with a window displacement of 66 TRs among the surrogate and actual connectome data (Mann-Whitney U test). $p < 0.05$ (two-sided, FDR corrected) is shown in **bold**.

	$^1G - {}^2R$	$^1T - {}^2R$	$^1GT - {}^2R$	$^1T - {}^2G$	$^1GT - {}^2G$	$^1GT - {}^2T$
p	**0.014**	$\mathbf{4.6 \times 10^{-3}}$	$\mathbf{4.2 \times 10^{-8}}$	0.62	$\mathbf{2.8 \times 10^{-3}}$	**0.018**
median_1	0.050	0.050	0.059	0.050	0.059	0.059
median_2	0.044	0.044	0.044	0.050	0.050	0.050
n_1	50	50	50	50	50	50
n_2	50	50	50	50	50	50
U	1619	1678	2069	1323	1706	1598
z-value	2.5	2.9	5.6	0.50	3.1	2.4
Cliff's delta	0.3	0.34	0.66	0.058	0.36	0.28
95% CI of delta	[0.07, 0.49]	[0.12, 0.53]	[0.46, 0.79]	[−0.17, 0.28]	[0.14, 0.55]	[0.05, 0.47]

	$^1\text{Actual} - {}^2R$	$^1\text{Actual} - {}^2G$	$^1\text{Actual} - {}^2T$	$^1\text{Actual} - {}^2GT$
p	$\mathbf{4.7 \times 10^{-17}}$	$\mathbf{1.3 \times 10^{-10}}$	$\mathbf{1.1 \times 10^{-8}}$	$\mathbf{2.0 \times 10^{-4}}$
median_1	0.070	0.070	0.070	0.070
median_2	0.044	0.050	0.050	0.059
n_1	100	100	100	100
n_2	50	50	50	50
U	4673	4174	3983	3475
z-value	8.7	6.7	5.9	3.9
Cliff's delta	0.87	0.67	0.59	0.39
95% CI of delta	[0.75, 0.93]	[0.50, 0.78]	[0.42, 0.72]	[0.20, 0.55]

Supplementary Table 8. Comparison of the SD of dynamic fluctuations in mean PC between the main and control RSN-constrained surrogate connectome data (Mann-Whitney U test). $p < 0.05$ ($z > 0$) and $p < 0.05$ ($z < 0$) (two-sided, FDR corrected) are shown in **bold** and ***bold italic***, respectively.

	CON	DMN	LIM	DAN	VAN	SMN	VIS
^{1}Main − ^{2}Control							
p	0.091	0.76	0.17	0.16	0.057	0.69	***4.0 × 10^−7***
median_1	0.066	0.063	0.064	0.061	0.065	0.061	0.059
median_2	0.063	0.063	0.060	0.064	0.060	0.062	0.066
n_1	100	100	100	100	100	100	100
n_2	100	100	100	100	100	100	100
U	5845	4873	5640	4308	5985	4780	2779
z-value	2.1	−0.31	1.6	−1.7	2.4	−0.54	−5.4
Cliff's delta	0.17	−0.025	0.13	−0.14	0.2	−0.044	−0.44
95% CI of delta	[0.01, 0.32]	[−0.18, 0.13]	[−0.03, 0.28]	[−0.29, 0.02]	[0.04, 0.35]	[−0.20, 0.12]	[−0.57, −0.29]
^{1}Main − ^2GT							
p	**1.9 × 10^−5**	**0.012**	**1.3 × 10^−3**	**0.018**	**7.7 × 10^−4**	**0.012**	0.36
median_1	0.066	0.063	0.064	0.061	0.065	0.061	0.059
median_2	0.057	0.057	0.057	0.057	0.057	0.057	0.057
n_1	100	100	100	100	100	100	100
n_2	50	50	50	50	50	50	50
U	3676	3158	3368	3109	3427	3182	2728
z-value	4.7	2.6	3.5	2.4	3.7	2.7	0.91
Cliff's delta	0.47	0.26	0.35	0.24	0.37	0.27	0.091
95% CI of delta	[0.28, 0.62]	[0.07, 0.44]	[0.15, 0.51]	[0.05, 0.42]	[0.18, 0.53]	[0.08, 0.44]	[−0.10, 0.28]
^{1}Control − ^{2}Actual							
p	***5.0 × 10^−3***	***5.0 × 10^−3***	***1.4 × 10^−4***	***0.011***	***5.3 × 10^−5***	***5.0 × 10^−3***	0.92
median_1	0.063	0.063	0.060	0.064	0.060	0.062	0.066
median_2	0.068	0.068	0.068	0.068	0.068	0.068	0.068
n_1	100	100	100	100	100	100	100
n_2	100	100	100	100	100	100	100
U	3803	3807	3316	3941	3167	3753	5043
z-value	−2.9	−2.9	−4.1	−2.6	−4.5	−3.0	0.10
Cliff's delta	−0.24	−0.24	−0.34	−0.21	−0.37	−0.25	8.6×10^{-3}
95% CI of delta	[−0.39, −0.08]	[−0.38, −0.08]	[−0.47, −0.18]	[−0.36, −0.05]	[−0.50, −0.21]	[−0.39, −0.09]	[−0.15, 0.17]

Supplementary Table 9. Comparison of the SD of dynamic fluctuations in mean TPC between the main and control RSN-constrained surrogate connectome data (Mann-Whitney U test). $p < 0.05$ ($z > 0$) and $p < 0.05$ ($z < 0$) (two-sided, FDR corrected) are shown in **bold** and ***bold italic***, respectively.

	CON	DMN	LIM	DAN	VAN	SMN	VIS
^{1}Main − ^{2}Control							
p	$\mathbf{4.1 \times 10^{-5}}$	0.10	$\mathbf{2.5 \times 10^{-5}}$	0.87	$\mathbf{1.3 \times 10^{-4}}$	0.067	$\boldsymbol{4.4 \times 10^{-15}}$
median_1	0.015	0.012	0.014	0.013	0.013	0.013	9.9×10^{-3}
median_2	0.013	0.012	0.012	0.013	0.012	0.012	0.014
n_1	100	100	100	100	100	100	100
n_2	100	100	100	100	100	100	100
U	6757	5703	6839	4935	6619	5811	1691
z-value	4.3	1.7	4.5	−0.16	4.0	2.0	−8.1
Cliff's delta	0.35	0.14	0.37	−0.013	0.32	0.16	−0.66
95% CI of delta	[0.19, 0.49]	[−0.02, 0.29]	[0.21, 0.50]	[−0.17, 0.15]	[0.17, 0.46]	[0.00, 0.31]	[−0.76, −0.53]
^{1}Main − ^2GT							
p	$\mathbf{3.1 \times 10^{-12}}$	$\mathbf{9.0 \times 10^{-7}}$	$\mathbf{1.6 \times 10^{-10}}$	$\mathbf{1.2 \times 10^{-7}}$	$\mathbf{3.7 \times 10^{-9}}$	$\mathbf{3.7 \times 10^{-9}}$	0.85
median_1	0.015	0.012	0.014	0.013	0.013	0.013	9.9×10^{-3}
median_2	9.7×10^{-3}	9.7×10^{-3}	9.7×10^{-3}	9.7×10^{-3}	9.7×10^{-3}	9.7×10^{-3}	9.7×10^{-3}
n_1	100	100	100	100	100	100	100
n_2	50	50	50	50	50	50	50
U	4317	3740	4152	3843	4005	4003	2453
z-value	7.2	4.9	6.6	5.4	6.0	6.0	−0.19
Cliff's delta	0.73	0.50	0.66	0.54	0.60	0.60	−0.019
95% CI of delta	[0.57, 0.83]	[0.31, 0.64]	[0.49, 0.78]	[0.36, 0.67]	[0.43, 0.73]	[0.43, 0.73]	[−0.21, 0.17]
^{1}Control − ^{2}Actual							
p	$\boldsymbol{7.8 \times 10^{-5}}$	$\boldsymbol{1.1 \times 10^{-7}}$	$\boldsymbol{2.5 \times 10^{-8}}$	$\boldsymbol{2.5 \times 10^{-3}}$	$\boldsymbol{1.1 \times 10^{-8}}$	$\boldsymbol{6.0 \times 10^{-6}}$	0.88
median_1	0.013	0.012	0.012	0.013	0.012	0.012	0.014
median_2	0.015	0.015	0.015	0.015	0.015	0.015	0.015
n_1	100	100	100	100	100	100	100
n_2	100	100	100	100	100	100	100
U	3350	2764	2632	3742	2530	3099	4938
z-value	−4.0	−5.5	−5.8	−3.1	−6.0	−4.6	−0.15
Cliff's delta	−0.33	−0.45	−0.47	−0.25	−0.49	−0.38	−0.012
95% CI of delta	[−0.47, −0.17]	[−0.57, −0.30]	[−0.60, −0.32]	[−0.40, −0.09]	[−0.61, −0.35]	[−0.51, −0.22]	[−0.17, 0.15]

Supplementary Table 10. Comparison of the SD of dynamic fluctuations in modularity between the main and control RSN-constrained surrogate connectome data (Mann-Whitney U test). $p < 0.05$ ($z > 0$) and $p < 0.05$ ($z < 0$) (two-sided, FDR corrected) are shown in **bold** and ***bold italic***, respectively.

	CON	DMN	LIM	DAN	VAN	SMN	VIS
^{1}Main − ^{2}Control							
p	**0.021**	0.83	0.10	0.65	$\mathbf{1.9 \times 10^{-3}}$	0.81	***9.6×10^{-7}***
median_1	0.033	0.031	0.032	0.032	0.033	0.031	0.030
median_2	0.032	0.031	0.031	0.033	0.030	0.031	0.034
n_1	100	100	100	100	100	100	100
n_2	100	100	100	100	100	100	100
U	6070	5088	5779	4702	6415	5160	2843
z-value	2.6	0.21	1.9	−0.73	3.5	0.39	−5.3
Cliff's delta	0.21	0.018	0.16	−0.060	0.28	0.032	−0.43
95% CI of delta	[0.05, 0.36]	[−0.14, 0.18]	[0.00, 0.31]	[−0.22, 0.10]	[0.12, 0.43]	[−0.13, 0.19]	[−0.56, −0.28]
^{1}Main − ^{2}*GT*							
p	$\mathbf{4.3 \times 10^{-7}}$	$\mathbf{1.3 \times 10^{-3}}$	$\mathbf{1.3 \times 10^{-4}}$	$\mathbf{1.6 \times 10^{-4}}$	$\mathbf{6.3 \times 10^{-6}}$	$\mathbf{1.3 \times 10^{-3}}$	0.16
median_1	0.033	0.031	0.032	0.032	0.033	0.031	0.030
median_2	0.028	0.028	0.028	0.028	0.028	0.028	0.028
n_1	100	100	100	100	100	100	100
n_2	50	50	50	50	50	50	50
U	3859	3320	3513	3483	3698	3330	2852
z-value	5.4	3.3	4.0	3.9	4.8	3.3	1.4
Cliff's delta	0.54	0.33	0.41	0.39	0.48	0.33	0.14
95% CI of delta	[0.36, 0.68]	[0.13, 0.49]	[0.21, 0.56]	[0.20, 0.55]	[0.29, 0.63]	[0.14, 0.50]	[−0.06, 0.32]
^{1}Control − ^{2}Actual							
p	***5.0×10^{-6}***	***1.6×10^{-6}***	***9.2×10^{-8}***	***6.9×10^{-4}***	***1.1×10^{-9}***	***9.2×10^{-8}***	0.12
median_1	0.032	0.031	0.031	0.033	0.030	0.031	0.034
median_2	0.035	0.035	0.035	0.035	0.035	0.035	0.035
n_1	100	100	100	100	100	100	100
n_2	100	100	100	100	100	100	100
U	3103	2992	2751	3593	2379	2745	4355
z-value	−4.6	−4.9	−5.5	−3.4	−6.4	−5.5	−1.6
Cliff's delta	−0.38	−0.40	−0.45	−0.28	−0.52	−0.45	−0.13
95% CI of delta	[−0.51, −0.22]	[−0.53, −0.25]	[−0.58, −0.30]	[−0.42, −0.12]	[−0.64, −0.38]	[−0.58, −0.30]	[−0.28, 0.03]

Supplementary Table 11. Comparison of the SD of dynamic fluctuations in mean PC with a window width of 44 TRs between the main and control RSN-constrained surrogate connectome data (Mann-Whitney U test). $p < 0.05$ ($z > 0$) and $p < 0.05$ ($z < 0$) (two-sided, FDR corrected) are shown in **bold** and ***bold italic***, respectively.

	CON	DMN	LIM	DAN	VAN	SMN	VIS
^{1}Main − ^{2}Control							
p	0.059	0.56	0.27	0.92	0.059	0.82	***1.6×10^{-8}***
median$_1$	0.070	0.066	0.067	0.066	0.067	0.064	0.059
median$_2$	0.067	0.066	0.063	0.067	0.063	0.066	0.068
n_1	100	100	100	100	100	100	100
n_2	100	100	100	100	100	100	100
U	5915	5346	5581	4956	5952	5156	2554
z-value	2.2	0.84	1.4	−0.11	2.3	0.38	−6.0
Cliff's delta	0.18	0.069	0.12	-8.8×10^{-3}	0.19	0.031	−0.49
95% CI of delta	[0.02, 0.33]	[−0.09, 0.22]	[−0.04, 0.27]	[−0.17, 0.15]	[0.03, 0.34]	[−0.13, 0.19]	[−0.61, −0.34]
^{1}Main − ^2GT							
p	**6.1×10^{-7}**	**9.5×10^{-4}**	**9.5×10^{-4}**	**1.2×10^{-3}**	**4.4×10^{-4}**	**1.2×10^{-3}**	0.99
median$_1$	0.070	0.066	0.067	0.066	0.067	0.064	0.059
median$_2$	0.060	0.060	0.060	0.060	0.060	0.060	0.060
n_1	100	100	100	100	100	100	100
n_2	50	50	50	50	50	50	50
U	3843	3368	3370	3322	3463	3334	2504
z-value	5.4	3.5	3.5	3.3	3.8	3.3	0.014
Cliff's delta	0.54	0.35	0.35	0.33	0.39	0.33	1.6×10^{-3}
95% CI of delta	[0.36, 0.67]	[0.15, 0.51]	[0.15, 0.51]	[0.13, 0.49]	[0.19, 0.54]	[0.14, 0.50]	[−0.19, 0.19]
^{1}Control − ^{2}Actual							
p	***6.5×10^{-4}***	***1.2×10^{-4}***	***1.0×10^{-5}***	***1.3×10^{-4}***	***1.2×10^{-6}***	***4.8×10^{-5}***	0.15
median$_1$	0.067	0.066	0.063	0.067	0.063	0.066	0.068
median$_2$	0.073	0.073	0.073	0.073	0.073	0.073	0.073
n_1	100	100	100	100	100	100	100
n_2	100	100	100	100	100	100	100
U	3587	3373	3086	3401	2862	3256	4414
z-value	−3.5	−4.0	−4.7	−3.9	−5.2	−4.3	−1.4
Cliff's delta	−0.28	−0.33	−0.38	−0.32	−0.43	−0.35	−0.12
95% CI of delta	[−0.42, −0.12]	[−0.46, −0.17]	[−0.52, −0.23]	[−0.46, −0.16]	[−0.56, −0.27]	[−0.49, −0.19]	[−0.27, 0.04]

Supplementary Table 12. Comparison of the SD of dynamic fluctuations in mean PC with a window width of 88 TRs between the main and control RSN-constrained surrogate connectome data (Mann-Whitney U test). $p < 0.05$ ($z > 0$) and $p < 0.05$ ($z < 0$) (two-sided, FDR corrected) are shown in **bold** and ***bold italic***, respectively.

	CON	DMN	LIM	DAN	VAN	SMN	VIS
[1]Main − [2]Control							
p	0.16	0.57	0.071	***0.033***	**0.015**	0.48	***1.4×10^{-6}***
median_1	0.065	0.061	0.061	0.058	0.062	0.060	0.056
median_2	0.062	0.062	0.059	0.062	0.058	0.061	0.066
n_1	100	100	100	100	100	100	100
n_2	100	100	100	100	100	100	100
U	5645	4766	5838	3995	6173	4664	2869
z-value	1.6	−0.57	2.0	−2.5	2.9	−0.82	−5.2
Cliff's delta	0.13	−0.047	0.17	−0.2	0.23	−0.067	−0.43
95% CI of delta	[−0.03, 0.28]	[−0.20, 0.11]	[0.01, 0.32]	[−0.35, −0.04]	[0.07, 0.38]	[−0.22, 0.09]	[−0.55, −0.27]
[1]Main − [2]*GT*							
p	**1.3×10^{-3}**	**0.0498**	**3.2×10^{-3}**	0.34	**3.2×10^{-3}**	0.086	0.75
median_1	0.065	0.061	0.061	0.058	0.062	0.060	0.056
median_2	0.057	0.057	0.057	0.057	0.057	0.057	0.057
n_1	100	100	100	100	100	100	100
n_2	50	50	50	50	50	50	50
U	3438	3050	3325	2765	3303	2970	2581
z-value	3.7	2.2	3.3	1.1	3.2	1.9	0.32
Cliff's delta	0.38	0.22	0.33	0.11	0.32	0.19	0.032
95% CI of delta	[0.18, 0.54]	[0.02, 0.40]	[0.14, 0.50]	[−0.09, 0.29]	[0.13, 0.49]	[−0.01, 0.37]	[−0.16, 0.22]
[1]Control − [2]Actual							
p	0.11	0.11	***6.3×10^{-3}***	0.11	***4.6×10^{-4}***	0.055	0.34
median_1	0.062	0.062	0.059	0.062	0.058	0.061	0.066
median_2	0.065	0.065	0.065	0.065	0.065	0.065	0.065
n_1	100	100	100	100	100	100	100
n_2	100	100	100	100	100	100	100
U	4257	4311	3722	4313	3365	4074	5392
z-value	−1.8	−1.7	−3.1	−1.7	−4.0	−2.3	0.96
Cliff's delta	−0.15	−0.14	−0.26	−0.14	−0.33	−0.19	0.078
95% CI of delta	[−0.30, 0.01]	[−0.29, 0.02]	[−0.40, −0.10]	[−0.29, 0.02]	[−0.47, −0.17]	[−0.33, −0.02]	[−0.08, 0.23]

Supplementary Table 13. Comparison of the SD of dynamic fluctuations in mean PC with a window displacement of 1 TR between the main and control RSN-constrained surrogate connectome data (Mann-Whitney U test). $p < 0.05$ ($z > 0$) and $p < 0.05$ ($z < 0$) (two-sided, FDR corrected) are shown in **bold** and ***bold italic***, respectively.

	CON	DMN	LIM	DAN	VAN	SMN	VIS
^{1}Main $-$ ^{2}Control							
p	0.11	0.76	0.11	0.11	0.064	0.76	$\boldsymbol{6.1 \times 10^{-7}}$
median_1	0.066	0.063	0.064	0.061	0.065	0.062	0.059
median_2	0.064	0.064	0.060	0.064	0.060	0.062	0.066
n_1	100	100	100	100	100	100	100
n_2	100	100	100	100	100	100	100
U	5818	4872	5713	4276	5966	4837	2809
z-value	2.0	-0.31	1.7	-1.8	2.4	-0.4	-5.4
Cliff's delta	0.16	-0.026	0.14	-0.14	0.19	-0.033	-0.44
95% CI of delta	$[0.00, 0.31]$	$[-0.18, 0.13]$	$[-0.02, 0.29]$	$[-0.30, 0.02]$	$[0.03, 0.34]$	$[-0.19, 0.13]$	$[-0.57, -0.29]$
^{1}Main $-$ ^{2}GT							
p	$\mathbf{2.8 \times 10^{-5}}$	**0.013**	$\mathbf{1.4 \times 10^{-3}}$	**0.025**	$\mathbf{1.1 \times 10^{-3}}$	**0.012**	0.35
median_1	0.066	0.063	0.064	0.061	0.065	0.062	0.059
median_2	0.057	0.057	0.057	0.057	0.057	0.057	0.057
n_1	100	100	100	100	100	100	100
n_2	50	50	50	50	50	50	50
U	3657	3152	3362	3078	3402	3181	2734
z-value	4.6	2.6	3.4	2.3	3.6	2.7	0.93
Cliff's delta	0.46	0.26	0.34	0.23	0.36	0.27	0.094
95% CI of delta	$[0.28, 0.61]$	$[0.06, 0.43]$	$[0.15, 0.51]$	$[0.03, 0.41]$	$[0.17, 0.52]$	$[0.08, 0.44]$	$[-0.10, 0.28]$
^{1}Control $-$ ^{2}Actual							
p	$\boldsymbol{7.2 \times 10^{-3}}$	$\boldsymbol{7.2 \times 10^{-3}}$	$\boldsymbol{1.9 \times 10^{-4}}$	***0.013***	$\boldsymbol{1.0 \times 10^{-4}}$	$\boldsymbol{6.1 \times 10^{-3}}$	0.85
median_1	0.064	0.064	0.060	0.064	0.060	0.062	0.066
median_2	0.068	0.068	0.068	0.068	0.068	0.068	0.068
n_1	100	100	100	100	100	100	100
n_2	100	100	100	100	100	100	100
U	3854	3847	3349	3964	3226	3768	5079
z-value	-2.8	-2.8	-4.0	-2.5	-4.3	-3.0	0.19
Cliff's delta	-0.23	-0.23	-0.33	-0.21	-0.35	-0.25	0.016
95% CI of delta	$[-0.38, -0.07]$	$[-0.38, -0.07]$	$[-0.47, -0.17]$	$[-0.36, -0.05]$	$[-0.49, -0.20]$	$[-0.39, -0.09]$	$[-0.14, 0.17]$

Supplementary Table 14. Comparison of the SD of dynamic fluctuations in mean PC with a window displacement of 66 TRs between the main and control RSN-constrained surrogate connectome data (Mann-Whitney U test). $p < 0.05$ ($z > 0$) and $p < 0.05$ ($z < 0$) (two-sided, FDR corrected) are shown in **bold** and ***bold italic***, respectively.

	CON	DMN	LIM	DAN	VAN	SMN	VIS
[1]Main − [2]Control							
p	0.50	0.58	0.58	0.50	0.070	0.58	***0.012***
median_1	0.066	0.065	0.063	0.061	0.068	0.062	0.061
median_2	0.065	0.064	0.060	0.063	0.059	0.063	0.067
n_1	100	100	100	100	100	100	100
n_2	100	100	100	100	100	100	100
U	5454	4769	5307	4563	5953	4776	3716
z-value	1.1	−0.56	0.75	−1.1	2.3	−0.55	−3.1
Cliff's delta	0.091	−0.046	0.061	−0.087	0.19	−0.045	−0.26
95% CI of delta	[−0.07, 0.25]	[−0.20, 0.11]	[−0.10, 0.22]	[−0.24, 0.07]	[0.03, 0.34]	[−0.20, 0.11]	[−0.40, −0.10]
[1]Main − [2]*GT*							
p	**0.025**	0.17	0.22	0.23	**0.045**	0.23	0.37
median_1	0.066	0.065	0.063	0.061	0.068	0.062	0.061
median_2	0.059	0.059	0.059	0.059	0.059	0.059	0.059
n_1	100	100	100	100	100	100	100
n_2	50	50	50	50	50	50	50
U	3230	2949	2886	2824	3125	2833	2726
z-value	2.9	1.8	1.5	1.3	2.5	1.3	0.90
Cliff's delta	0.29	0.18	0.15	0.13	0.25	0.13	0.090
95% CI of delta	[0.10, 0.46]	[−0.02, 0.36]	[−0.04, 0.34]	[−0.07, 0.31]	[0.05, 0.42]	[−0.06, 0.32]	[−0.10, 0.28]
[1]Control − [2]Actual							
p	***0.036***	0.059	***1.9×10^{-3}***	***0.036***	***5.3×10^{-4}***	***0.036***	0.74
median_1	0.065	0.064	0.060	0.063	0.059	0.063	0.067
median_2	0.070	0.070	0.070	0.070	0.070	0.070	0.070
n_1	100	100	100	100	100	100	100
n_2	100	100	100	100	100	100	100
U	4007	4200	3586	4086	3380	4079	4863
z-value	−2.4	−2.0	−3.5	−2.2	−4.0	−2.2	−0.33
Cliff's delta	−0.20	−0.16	−0.28	−0.18	−0.32	−0.18	−0.027
95% CI of delta	[−0.35, −0.04]	[−0.31, 0.00]	[−0.43, −0.12]	[−0.33, −0.02]	[−0.46, −0.17]	[−0.33, −0.02]	[−0.18, 0.13]

Supplementary Table 15. Comparison of the SD of dynamic fluctuations in global network measures for segregation and integration between the simulated data (with the actual connectome) and empirical data (Mann-Whitney U test, two-sided).

	Mean PC	Mean TPC	Modularity
^{1}Empirical − ^{2}Simulated (Actual)			
p	7.5×10^{-15}	1.7×10^{-11}	7.3×10^{-22}
median_1	0.081	0.018	0.044
median_2	0.068	0.015	0.035
n_1	336	336	336
n_2	100	100	100
U	25401	24242	27430
z-value	7.8	6.7	9.6
Cliff's delta	0.51	0.44	0.63
95% CI of delta	$[0.40, 0.61]$	$[0.32, 0.55]$	$[0.53, 0.72]$

Mean PC	Window width		Window displacement	
	44 TRs	88 TRs	1 TR	66 TRs
^{1}Empirical − ^{2}Simulated (Actual)				
p	2.5×10^{-7}	4.8×10^{-14}	5.8×10^{-15}	6.9×10^{-10}
median_1	0.078	0.080	0.081	0.084
median_2	0.073	0.065	0.068	0.070
n_1	336	336	336	336
n_2	100	100	100	100
U	22503	25139	25438	23623
z-value	5.2	7.5	7.8	6.2
Cliff's delta	0.34	0.50	0.51	0.41
95% CI of delta	$[0.21, 0.45]$	$[0.38, 0.60]$	$[0.40, 0.61]$	$[0.28, 0.51]$
