# Supplementary material for: Structural determinants of dynamic fluctuations between segregation and integration on the human connectome
Source: Commun Biol. 2020 Oct 23;3:606. doi: 10.1038/s42003-020-01331-3 (PMC7584581; doi:10.1038/s42003-020-01331-3)
Supplement: Supplementary file 3 — Description of Additional Supplementary Files [file 42003_2020_1331_MOESM3_ESM.docx]

**Description of Additional Supplementary Files**

Supplementary Data 1: Numerical data sets (in Excel spreadsheet form) that underlie Figs. 1 and 3-5 and Supplementary Figs. 1-14
